# Progress of nanoparticle drug delivery system for the treatment of glioma

**DOI:** 10.3389/fbioe.2024.1403511

**Published:** 2024-06-11

**Authors:** Guogang Lai, Hao Wu, Kaixia Yang, Kaikai Hu, Yan Zhou, Xiao Chen, Fan Fu, Jiayi Li, Guomin Xie, Hai-Feng Wang, Zhongyue Lv, Xiping Wu

**Affiliations:** ^1^ Department of Neurology, The Affiliated Lihuili Hospital of Ningbo University, Ningbo, Zhejiang, China; ^2^ Ningbo Institute of Innovation for Combined Medicine and Engineering, The Affiliated Lihuili Hospital of Ningbo University, Ningbo, Zhejiang, China

**Keywords:** glioma, nanoparticle drug delivery systems, nanoparticle, blood-brain barrier, brain targeting

## Abstract

Gliomas are typical malignant brain tumours affecting a wide population worldwide. Operation, as the common treatment for gliomas, is always accompanied by postoperative drug chemotherapy, but cannot cure patients. The main challenges are chemotherapeutic drugs have low blood-brain barrier passage rate and a lot of serious adverse effects, meanwhile, they have difficulty targeting glioma issues. Nowadays, the emergence of nanoparticles (NPs) drug delivery systems (NDDS) has provided a new promising approach for the treatment of gliomas owing to their excellent biodegradability, high stability, good biocompatibility, low toxicity, and minimal adverse effects. Herein, we reviewed the types and delivery mechanisms of NPs currently used in gliomas, including passive and active brain targeting drug delivery. In particular, we primarily focused on various hopeful types of NPs (such as liposome, chitosan, ferritin, graphene oxide, silica nanoparticle, nanogel, neutrophil, and adeno-associated virus), and discussed their advantages, disadvantages, and progress in preclinical trials. Moreover, we outlined the clinical trials of NPs applied in gliomas. According to this review, we provide an outlook of the prospects of NDDS for treating gliomas and summarise some methods that can enhance the targeting specificity and safety of NPs, like surface modification and conjugating ligands and peptides. Although there are still some limitations of these NPs, NDDS will offer the potential for curing glioma patients.

## Graphical Abstract

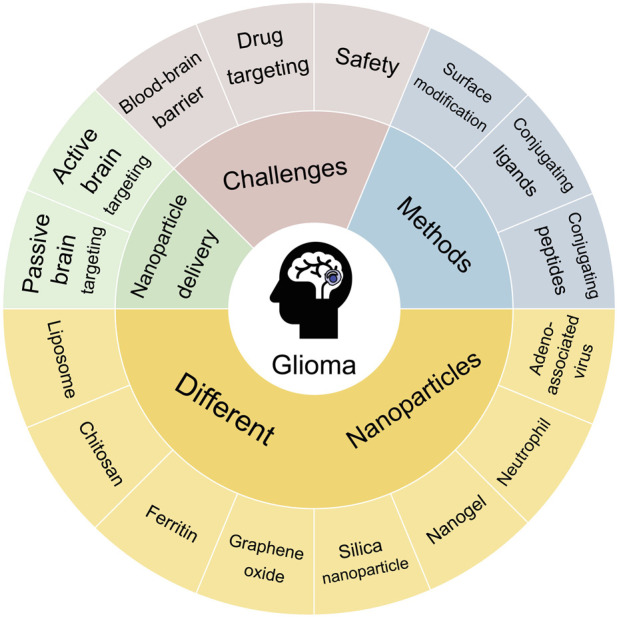

## 1 Introduction

**FIGURE 1:**
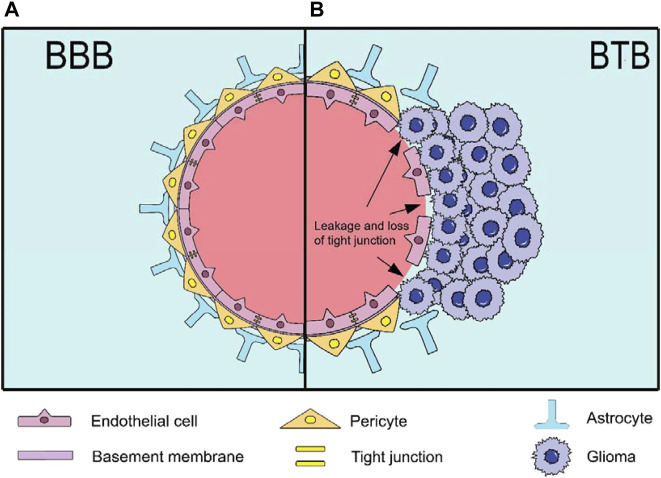
The structure of BBB and BTB. **(A)** The BBB is a highly selective and low-permeability barrier between the blood and the brain. It has a capillary wall formed by endothelial cells (ECs) and tight junction proteins inside, while the outside is surrounded by pericytes, basement membrane and astrocytes; **(B)** The BTB is formed by gliomas after destroying the BBB. The main changes and disruption include detached astrocytes, loss of tight junctions (resulting in leakage) and the destroyed basement membrane.

Gliomas are among the most prevalent malignant brain tumours and originate from glial cells, constituting approximately 80%–85% of adult brain tumours. In 2021, the World Health Organization (WHO) updated its classification of tumours to include molecular changes and the latest understanding of pathophysiology. It identified diffuse glioma as the most common and aggressive type of adult glioma, categorised into (1) astrocytoma, IDH-mutant (WHO grades 2–4); (2) oligodendroglioma, IDH-mutant and 1p/19q co-deleted (WHO grades 2 and 3); (3) glioblastoma (GBM), IDH-wildtype (WHO grade 4) (Berger et al., 2022). Similarly, its incidence is high, at approximately seven per 100,000 people, and tends to increase with age. Unfortunately, the 5-year survival rate is approximately 36%. The current standard therapies for patients primarily involve surgery, radiotherapy, and chemotherapy (Wen et al., 2020; Zhao et al., 2020; Schaff and Mellinghoff, 2023). However, the invasive nature of gliomas results in extensive tumour infiltration (Khan et al., 2022), making complete surgical resection unfeasible in most cases, particularly in glioblastomas, which are prone to relapse. Moreover, the survival extension achieved through combination of radiotherapy and chemotherapy post-surgery is only 2–4 months (Khan et al., 2022). Importantly, many anti-tumour drugs face challenges in bypassing the blood-brain barrier (BBB) owing to its high selectivity and low permeability, hindering the passage of almost all macromolecules and 98% of small molecules (Figure 1) (Terstappen et al., 2021). Additionally, the safety and targeted delivery of these drugs warrant attention (Meng W et al., 2023; Ramalho MJ et al., 2023). There is an urgent need to develop novel therapeutic strategies to overcome these challenges.

In recent years, nanoparticle (NP) drug delivery systems (NDDS) based on NPs have received widespread attention owing to their excellent biodegradability, high stability, good biocompatibility, low toxicity, and minimal side effects (Nance et al., 2014; Zhang T. T. et al., 2016; Janjua et al., 2021b). It is noteworthy that gliomas form a blood-tumour barrier (BTB) after BBB disruption (Figure 1) (Mojarad-Jabali et al., 2021). The BTB is not only considered more permeable than the BBB, facilitating NP penetration, but also exhibits an enhanced permeability and retention (EPR) effect, leading to the passive accumulation of NPs at the tumour site (Sun et al., 2022; Zi et al., 2022). Moreover, it blocks lymphatic reflux in the tumour area, enabling NPs to remain in the tumour tissue for an extended period and prolonging the efficacy of the drug (Fang et al., 2011; Shi et al., 2017). Interestingly, NPs can leverage their inherent characteristics or undergo surface modifications to achieve active brain targeting drug delivery. Advancements in NDDS have produced various nanomaterials for glioma treatment, including but not limited to lipid-based (Kim SS et al., 2015; Ashrafzadeh et al., 2020; Ghaferi et al., 2022), polymeric (Ahmadi Nasab et al., 2018; Turabee et al., 2019; Alswailem et al., 2022), protein-based (Zhang J. et al., 2021), carbon-based (Song et al., 2017), inorganic (Janjua et al., 2021a), nanogel (NG) (Zhang M. et al., 2021), cell-based (Xue et al., 2017; Li Y. et al., 2021; Wang J. et al., 2021) and virus-based NPs (Crommentuijn et al., 2016; GuhaSarkar et al., 2016). However, owing to the limitations and drawbacks of different NPs, there are currently no clinical drugs specifically used to treat gliomas, although some have been approved for other diseases (Table 1) (Barenholz, 2012; Murata and Teshima, 2021; Blair, 2022; Keam, 2022; Heo, 2023).

**TABLE 1:** The classification of NDDS and the advantages and disadvantages of delivery carriers in the treatment of glioma.

Nanoparticle delivery systems	Carriers		Polymers	Carriers’ advantages	Carriers’ disadvantages	Ref.
Lipid-based nanoparticles	Lip		PEG-Lip-DOX/CB	1. Good biocompatibility 2. Good biodegradability 3. Low toxicity 4. hydrophilic and lipophilic properties	1. The physical and chemical instability 2. The low loading 3. The high costs of mass production	Ghaferi et al. (2022)
			PEG-Lip-TMZ			Waghule et al. (2023a)
			PTX-R8-dGR-Lip			Liu et al. (2016)
			Tf-LPs			Wang et al. (2019)
Polymeric nanoparticles	CS		PF127-TMC/DTX	1. Good biocompatibility 2. Good biodegradability 3. No or low toxicity 4. Low cost	1. A risk of causing thrombosis 2. Poor water solubility 3. Poor endosomal escape ability 4. The safety issues in the human body need further evaluation	Turabee et al. (2019)
			CS-microRNA-219			Alswailem et al. (2022)
			Cur@CS-MCM-41			Ahmadi Nasab et al. (2018)
Protein-based nanoparticles	Ferrin	HFN	HFN-PTX	1. Uniform size 2. Low immunogenicit 3. Good biocompatibility 4. pH-responsive drug release capability 5. Self-assembly to form nanocage structures	1. The competitive binding from the endogenous Tf 2. TfR has a bidirectional trans-cellular function 3. TfR receptors are present in multiple tissues and organs	Zhang et al. (2021b)
Carbon-based nanoparticles	GO		Lf@GO@Fe3O4@DOX	1. Good biocompatibility 2. Large specific surface area conducive to biomolecule modification 3. High drug loading capacity 4. Relatively low toxicity	1. In the early stages of research 2. The safety of modified GO *in vivo* requires further research confirmation 3. The technology for drug release control is not yet mature enough	Song et al. (2017)
			GO-FA-TMZ			Wang et al. (2020)
Inorganic nanoparticles	SiNPs		SiNPs	1. Abundant inorganic compounds 2. Uniform pore size 3. Easy to surface modification 4. Good biocompatibility 5. stability	1. Leading to oxidative stress 2. Mitochondrial dysfunction 3. Activation of inflammatory responses 4. Causing cell death	Kusaczuk et al. (2018)
			USLP-PO3-DOX-Lf or USLP-NH2-DOX-Lf			Janjua et al. (2021a)
Nanogel	Nanogel		Lf-DOX/PBNG	1. High biocompatibility 2. excellent drug-loading capacity 3. responsiveness to environmental stimuli	1. Immunogenicity 2. Colloidal instability	Zhang et al. (2021c)
			ARNGs@TMZ/ICG			Zhang et al. (2022)
Cell-based nanoparticles	Leucocyte	NEs	NEs-Exos/DOX	1. Large quantity in the human body 2. First recruited to the tumor or inflammatory sites 3. Human neutrophils are not immunogenic 4. Good biocompatibility	1. It is easy to cause inflammatory reaction after large input 2. Exogenous NEs can easily lead to immune responses 3. The extraction and purification are very difficult 4. High cost	Wang et al. (2021b)
			ZGO@TiO2@ALP-NEs			Li et al. (2021a)
			PTX-CL/NEs			Xue et al. (2017)
Virus-based nanoparticles	adeno-associated virus	AAV9	AAV9-CBA-sTRAIL or AAV9-NSE-sTRAIL	1. High delivery efficiency 2. No pathogenicity 3. CNS cell tropism	1. Cause inflammatory responses and immune responses 2. Broad transduction effect lacks Specificity 3. Small packaging capacity 4. High cost	Crommentuijn et al. (2016)
			AAV9-IFN-β			GuhaSarkar et al. (2016)

Abbreviation: EPR, effect, the enhanced permeability and retention effect; Lip, liposome; PEG, polyethylene glycol(PEG)ylated; DOX, doxorubicin; CB, carboplatin; TMZ, temozolomide; PTX, paclitaxel; R8, a type of cell-penetrating peptides (made up of eight arginines, RRRRRRRR); dGR, an RGD (RGDfK, a type of CPP) reverse sequence (lower case letter represents D-amino acid residue); Tf-LPs, R8 and transferrin co-modified DOX-loaded Lip; CS, chitosan; PF127: pluronic F127; TMC: N,N,N-trimethyl chitosan; DTX, docetaxel; Cur, curcumin; MCM-41, mobil composition of matter No. 41, a type of MSNs; TfR, transferrin acceptor; GO, graphene oxide; Lf, lactoferrin; FA, folic acid; SiNPs, silica nanoparticles; USLP, ultra-small, large pore silica nanoparticles; -PO3, phosphate surface functionalization; -NH2, amine surface functionalization; PBNG, PBA, functionalized nanogels (PBA, phenylboronic acid); ARNGs, nanogel camouflaged with apolipoprotein E peptide-decorated erythrocyte membranes; ICG, indocyanine green; NEs, neutrophils; Exos, exosomes; ALP, anti-PD-1, antibody with PTX, loaded liposome; CL, cationic liposomes; AAV9, adeno-associated virus 9; IFN-β, interferon-β.

In this review, we discussed the delivery mechanisms of NDDS, encompassing passive brain targeting drug delivery utilising the EPR effect, as well as adsorption, receptor, cell, and virus-mediated active brain targeting drug delivery. Special attention is given to discussing the advantages and disadvantages of different types of NPs, along with the progress of preclinical trials. Additionally, an overview of the current clinical trials involving related carriers is provided. Collectively, the emerging treatment strategy for NDDS holds promise in offering new hope to patients with glioma.

## 2 The delivery mechanisms of nanoparticle drug delivery systems

Currently, the delivery mechanisms of NDDS include, paracellular pathways, passive diffusion, pathways utilising the EPR effect, adsorption-mediated transcytosis (AMT), receptor-mediated transcytosis (RMT), cell-mediated transcytosis (CMT), and viral-mediated transcytosis. Notably, the first three are associated with passive brain targeting drug delivery, whereas the remaining methods are considered for active brain targeting drug delivery. In this review, we examined these delivery mechanisms and preclinical trials of various carriers that utilise these mechanisms.

### 2.1 Passive brain targeting drug delivery

#### 2.1.1 Paracellular pathways and passive diffusion

**FIGURE 2:**
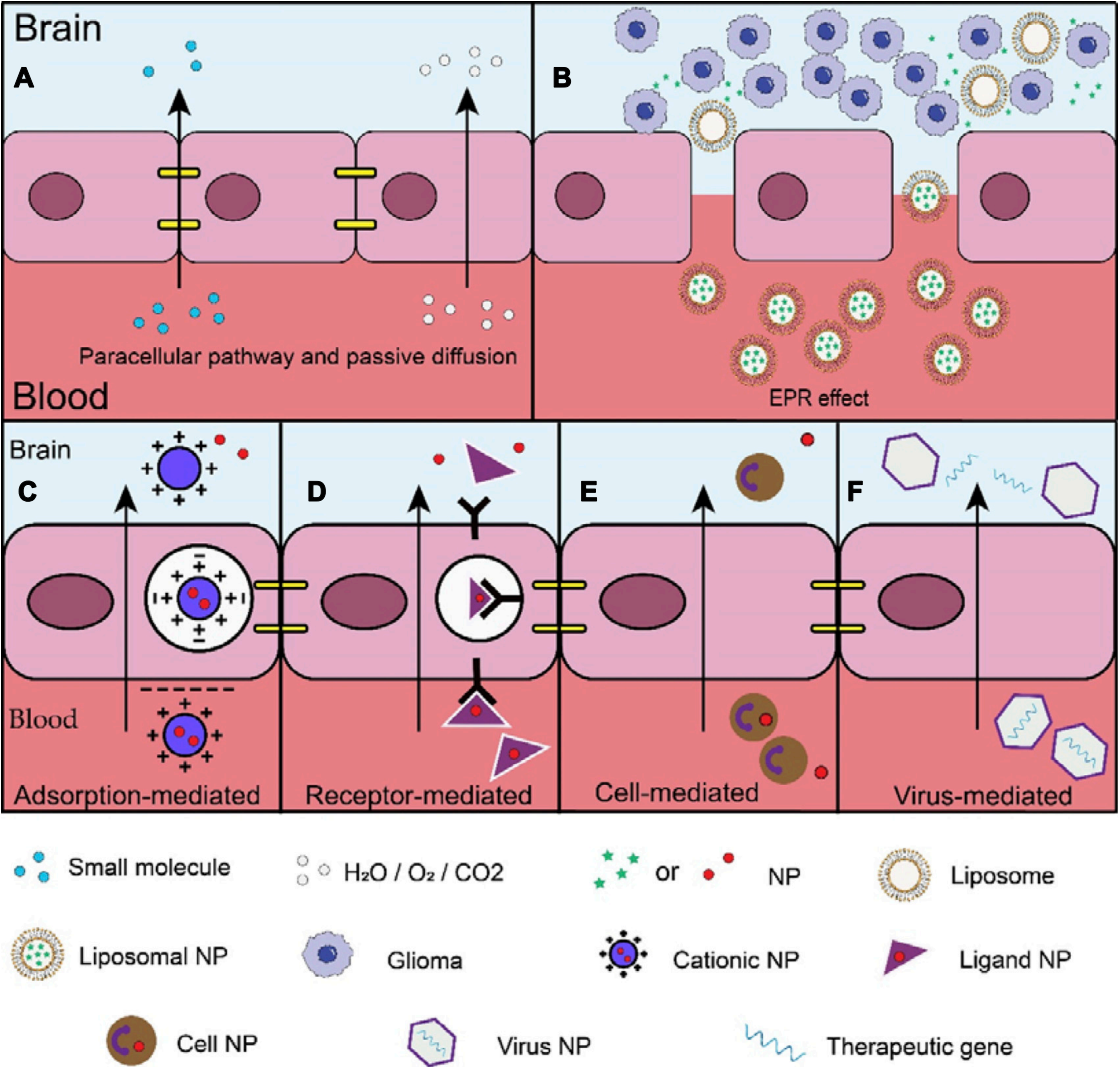
Different mechanisms of passive and active brain targeting drug delivery. **(A)** The tight junctions between endothelial cells only allow 2% of small molecules to enter the brain through paracellular pathways, the BBB's semi-permeability only allows oxygen, carbon dioxide, and water to enter through passive diffusion; **(B)** The large gaps between tumor vascular endothelial cells allow nanomaterials to enter tumor tissues. NPs can also stay in the tumor tissue for a long time, prolonging the functioning time of drugs; **(C)** Adsorption-mediated (AMT) transcytosis: the main mechanism is the electrostatic interaction of cations and anions; **(D)** Receptor-mediated transcytosis (RMT): the main mechanism is through the combination of ligands and specific receptors; **(E)** Cell-mediated transcytosis (CMT): the main mechanism depends on the characteristics of the cell itself; **(F)** Virus-mediated transcytosis: the mechanism crossing the BBB is not well understood, possibly by RMT or by other means.

Under physiological conditions, substances can enter the brain through passive transport, including paracellular pathways and passive diffusion (Figure 2). However, tight junctions between endothelial cells prevent 98% of the small molecules and all large molecules from entering the brain via paracellular pathways (Khalil et al., 2023). Additionally, the semi-permeability of the BBB allows only oxygen, carbon dioxide, and water to enter through passive diffusion (Khalil et al., 2023). Overall, this method is unsuitable under normal physiological conditions.

#### 2.1.2 Enhanced permeability and retention effect

Interestingly, the EPR effect is a unique phenomenon in tumour tissues (Figure 2). This effect is rooted in the distinctive anatomical and pathophysiological features of tumours, where the wide gaps between tumour vascular endothelial cells enable the entry of nanomaterials into the tumour tissues (Fang et al., 2020; Zi et al., 2022). Furthermore, the blocked lymphatic reflux in the tumour area allows NPs to remain in the tumour tissue for an extended period, thereby prolonging drug efficacy (Fang et al., 2011; Shi et al., 2017). Therefore, the EPR effect has been closely linked to nearly all NDDS used in tumour therapy over the past 30 years (Moosavian et al., 2021; Sun et al., 2022). Notably, the EPR effect varies between different tumours, leading to heterogeneity (Liu and Xu, 2022). Additionally, the size, shape, softness, and surface properties of the NPs can directly or indirectly influence the EPR effect (Ikeda-Imafuku M et al., 2022). For example, NPs within the range of 12–200 nm can effectively target tumour sites (Caro et al., 2021), while those below 12 nm are cleared by the kidney and those above 200 nm are cleared by a single phagocytic system (Caro et al., 2021). Studies have shown that spherical NPs accumulate the most in breast cancer, while rod-shaped NPs accumulate most rapidly in lung cancer (Black KC et al., 2014; Zhang L. et al., 2019). Moreover, softer NPs exhibit greater tumour accumulation and penetration (Liang et al., 2019). However, cationic NPs can form aggregates with anionic proteins in the blood, leading to a weakened EPR effect (Qi et al., 2011). While the surface charge of most NPs does not appear to affect the EPR effect, in some cases, it may affect passive targeting based on EPR effects (Ikeda-Imafuku M et al., 2022). Therefore, the selection of suitable NPs should be based on the characteristics of the EPR effect in gliomas.

### 2.2 Active brain targeting drug delivery

Although passive targeting offers significant advantages in tumour treatment, not all therapeutic doses can effectively reach the target tissues, and some drugs may leak into normal tissues. The significance of the EPR effect in tumour-targeted NP drug treatment has been a subject of controversy for many years (Shi et al., 2020). Notably, research has revealed that nearly all NPs (approximately 97%) are transported to tumour sites via active transport rather than the EPR effect (de Lazaro and Mooney, 2020; Sindhwani et al., 2020). Consequently, active targeting has been increasingly used to enhance drug delivery, thereby improving the precision and safety of targeting.

Compared with passive brain targeting drug delivery, active brain targeting drug delivery exhibits greater selectivity. This is exemplified by adsorption-mediated transcytosis (involving electrostatic interactions between cations and anions) and receptor-mediated transcytosis (involving the binding of ligands to specific receptors). Furthermore, active brain targeting drug delivery demonstrates a stronger affinity, as observed in cell and virus-mediated transcytosis. Active brain targeting drug delivery can efficiently transport NP drugs to the intended site, leading to enhanced therapeutic effects and reduced local cytotoxicity (Kang et al., 2018). Overall, these advantages establish active brain targeting drug delivery as a reliable strategy (Figure 2).

#### 2.2.1 Adsorption-mediated transcytosis

Researchers have extensively investigated the AMT as a primary pathway for active brain targeting drug delivery (Song et al., 2021). The principal mechanism involves electrostatic interactions between cations and anions. Specifically, cationic NPs and anionic endothelial cell membrane surfaces form strong adsorption via electrostatic interactions, leading to the formation of transcytotic vesicles (Liu and Xu, 2022). Subsequently, these vesicles fuse with the cell membrane and transport the NPs into the brain (Liu and Xu, 2022). Consequently, various NPs have been directly coated with cationic molecules on their surfaces, such as CS (Yu et al., 2019; Narmani and Jafari, 2021), or modified with cationic molecules on polymers, such as Cell-penetrating peptides (CPPs) (Lu et al., 2020; Gravina et al., 2022), enabling them to traverse the BBB via this route.

#### 2.2.2 Receptor-mediated transcytosis

RMT is a promising strategy for the active targeting of drug delivery to the brain. The primary mechanism involves the binding of ligands to specific receptors. Compared with adsorptive-mediated endocytosis (AMT), the cationic surface of AMT may lead to increased cytotoxicity, and its electrostatic interactions are non-specific (Song et al., 2021). Therefore, RMT has higher safety, specificity and selectivity. In recent years, numerous receptors, including Tf (TfRs) (Liu et al., 2020; Sun et al., 2020; Ramalho et al., 2022a), Lf (LfRs) (Song et al., 2017; Janjua et al., 2021a; Zhang M. et al., 2021), insulin (IRs) (Kuo and Shih-Huang, 2013), lipoprotein receptor (LPRs) (Wang R. et al., 2022; Ismail et al., 2022) have been found to be expressed in the BBB and brain tumour cells (Ouyang et al., 2022; Qiu et al., 2022). Utilising specific ligands for these receptors for drug delivery is beneficial. In this context, we will primarily focus on Tf and Lf.

#### 2.2.3 Cell-mediated transcytosis

CMT is a promising therapeutic approach that leverages a large number of cells in the human body. Certain cells can serve as drug carriers capable of traversing the BBB, earning them the moniker “Trojan horse cells” (Ayer and Klok, 2017), with examples including red blood cells, leukocytes, and stem cells. This delivery method is characterised by the inherent ability of carrier cells to circulate for an extended period, thereby prolonging the half-life of the drugs (Azarmi et al., 2020). Moreover, compared with alternative delivery methods, CMT exhibits lower cytotoxicity and immunogenicity, effectively mitigating adverse reactions in the body (Yu et al., 2020; Pawar et al., 2022). Herein, we primarily focused on leukocytes. Other drug delivery carriers based on different cell types that are not covered here, can be found in other reviews (Li Z. et al., 2021; Yang et al., 2022; Chao et al., 2023; Chen et al., 2023).

#### 2.2.4 Viral-mediated transcytosis

Currently, the mechanisms by which viruses act as carriers to cross the BBB, possibly through RMT or other means, are not well understood (Chen et al., 2021; Liu et al., 2021). Viruses possess the unique ability to bind to and enter host cells, thereby evading the host’s immune system. Upon entering the host cell and releasing the viral genome, the virus undergoes reverse transcription in a specific manner to replicate (Loo et al., 2021; Qiu et al., 2022). Therefore, drug delivery using viruses has garnered significant interest. Over the past two decades, viruses have been used as carriers for gene therapy in gliomas (Mozhei et al., 2020). However, serious adverse effects, including rare cases of treatment-related mortality (Bulcha et al., 2021), can occur owing to immunogenicity (Hamilton and Wright, 2021; Muhuri et al., 2021). Therefore, the safety of viral carriers must be thoroughly assessed before they can be used. Current viral carriers include adenoviruses (Ads), AAVs, and lentiviruses (LVs) (Pena et al., 2020), with AAVs receiving the greatest attention. Therefore, our focus was primarily on AAVs.

## 3 Pre-clinical progress

### 3.1 Lipid-based nanoparticles

Similar to the physiological structure of the cell membrane, the Lip is composed of a water nucleus surrounded by a lipid bilayer and exhibits good biocompatibility, biodegradability, low toxicity, and hydrophilic and lipophilic properties (Nakhaei et al., 2021; Lombardo and Kiselev, 2022). Consequently, they are among the most common carriers for passive targeting delivery of drugs. However, a key challenge faced by Lips is their physical and chemical instability, which can lead to adverse reactions and uncertainty regarding their efficacy (Nakhaei et al., 2021). Importantly, the stability of Lips can be effectively improved through polyethylene glycol (PEG)ylation (Najlah et al., 2019). For instance, Ghaferi et al. prepared PEGylated Lip (PEG-Lip) NP loaded with doxorubicin (DOX) and carboplatin (CB), which demonstrated superior therapeutic effects, safety, extended survival times, and enhanced drug loading compared with Lip-DOX/CB and DOX + CB (Ghaferi et al., 2022). Waghule et al. formulated and investigated PEGylated lipid nanocarriers loaded with temozolomide (TMZ), referred to as PEG-Lip-TMZ. In comparison with free TMZ, both Lip-TMZ and PEG-Lip-TMZ exhibited increased cytotoxicity, with enhancements of 1.7-fold and 1.6-fold, respectively. Moreover, PEGylated NPs provided a longer blood circulation time, with a 1.25-fold increase compared to that of Lip-TMZ (Waghule et al., 2023b). Cationic CPPs demonstrate superior cell membrane penetration capabilities (Zhou et al., 2022), so they can traverse the BBB through electrostatic interactions with negatively charged proteins on the cell membrane, making them the most commonly used (Zhou et al., 2022). For instance, R8 (comprising eight arginine residues, RRRRRRRR) is one of the most common traditional CPPs (Tian and Zhou, 2021). In order to improve the targeting, Liu et al. demonstrated that an RGD reverse sequence dGR was conjugated to R8 (R8-dGR), which could bind to both integrin αvβ3 and neuropilin-1 receptors. When applied to Lips to load paclitaxel (PTX-R8-dGR-Lip), this modification effectively targeted gliomas and improved penetration (Liu et al., 2016). The TfR has been reported to be expressed on both the BBB and tumour cells (Ramalho et al., 2022b). Importantly, TfR may be overexpressed in tumour cells up to 100 times more than in normal cells, as tumour cells require a large amount of iron for rapid proliferation (Choudhury et al., 2018). Therefore, Tf can be used to treat gliomas via specific receptor binding. Wang et al. developed R8 and Tf co-modified DOX-loaded liposomes (Tf-LPs), aiming to efficiently target glioma and overcome the BBB (Wang et al., 2019). Their team discovered that glioma cells effectively took up Tf-LPs. Compared to free DOX and LPs, Tf-LPs exhibited potent and low-toxicity anti-tumour effects. Therefore, Tf-LPs may be worth further study.

Although PEGylation can improve the stability of Lips, it is still necessary to address the low loading of Lip NPs (owing to lipid bilayer thickness limitations) and high cost of mass production (De Leo et al., 2022; Lombardo and Kiselev, 2022). Furthermore, the EPR effect observed in animal models may not directly apply to humans (Zi et al., 2022). Thus, additional verification is required to determine whether the use of Lip or PEG-Lip provides clinical benefits to patients. Notably, numerous clinical trials have demonstrated the viability of this carrier delivery method, and further details are discussed in the subsequent clinical trial section. Additionally, the targeting of Lips can be enhanced through conjugating ligands and/or peptides. Overall, Lips, particularly PEG-Lips, are a promising option.

### 3.2 Polymeric nanoparticles

CS is a naturally occurring amino acid polysaccharide known for its biocompatibility, biodegradability, low toxicity, and cost-effectiveness (Eslahi et al., 2021; Narmani and Jafari, 2021). Its cationic properties contribute to its water solubility and bioadhesion, enabling excellent binding affinity with negatively charged brain endothelial cells (BECs) (Mikusova and Mikus, 2021). Alswailem et al. developed microRNA-219 (miR-219) loaded CS NPs (CNPs), that inhibited tumour growth by regulating gene expression in GBM (Alswailem et al., 2022). This study demonstrated that the cationic properties of CS not only facilitate BBB penetration but also form complexes with anionic miR-219, enhancing its stability. Moreover, the CNPs loaded with miR-219 exhibited specificity for GBM cells, satisfying the drug targeting and safety requirements (Alswailem et al., 2022). Nasab et al. utilised CS-modified mesoporous silica to load curcumin, thereby creating Cur@CS-MCM-41. This modification reduced the potential toxicity of unmodified MCM-41 to cell membranes. *In vitro* experiments revealed that CS-MCM-41 not only enabled sustained release and increased the solubility of curcumin but also enhanced its bioavailability and toxic effects on GBM cells (Ahmadi Nasab et al., 2018). Moreover, primary amino group of CS exhibits sensitive stimuli-responsive characteristics, such as pH and temperature responsiveness, ensuring targeted drug delivery at specific times and doses (Budiarso et al., 2023). Turabee et al. developed a thermosensitive hydrogel drug delivery system using Pluronic F127 (PF127) and N,N,N-trimethyl CS (TMC) to load docetaxel, known as PF127-TMC/DTX. The addition of TMC increased the number of pores in the gel network, leading to enhanced drug release. PF127-TMC/DTX exhibited sustained drug release over 30 days and demonstrated greater growth inhibition of GBM cells than free DTX and PF127/DTX (Turabee et al., 2019). Thus, CS-based NPs are promising drug delivery systems for glioma treatment.

These studies underscore the exceptional potential of CS in diverse drug delivery applications owing to its advantageous properties. However, ongoing research is focused on enhancing the properties of CS further (Mikusova and Mikus, 2021). For example, CS exhibits poor water solubility and limited endosome escape ability, necessitating structural modifications (Zhang E. et al., 2019). Additionally, blood compatibility of CS is not promising and carries the risk of thrombosis (Sachdeva et al., 2023). Moreover, although CS has demonstrated low or non-toxicity in animal experiments, further studies are required to confirm its toxicity in humans (Zhang E. et al., 2019).

### 3.3 Protein-based nanoparticles

Ferrin is found universally across all living organisms and is composed of heavy chain ferrin (HFN) and light chain ferrin (LFN). It serves as an endogenous protein responsible for transporting and storing iron, thereby regulating the dynamic balance of Fe(II) and Fe(III) in the human body (Zhang B. et al., 2021). Additionally, ferrin has the advantages of uniform size, low immunogenicity, good biocompatibility, pH-responsive drug release capability, and self-assembly to form nanocage structures (Xia et al., 2024).

Similar to transferrin, ferrin can bind to TfR1 on endothelial and tumour cells. Liu et al. developed endogenous human ferritin heavy chain nanocages (HFn) for the specific delivery of PTX to the target by binding to TfR1. Satisfactory targeting effects were observed, which promoted PTX accumulation in the brain. HFn-PTX exhibited potent anti-GBM effects and outperformed free PTX. Importantly, no competitive inhibition was observed between HFn and endogenous Tf (Liu et al., 2020). However, the preparation conditions for HFn are challenging, and the process of NP formation in the cavity is sensitive to the reaction conditions (Zhang J. et al., 2021). Therefore, its potential for widespread application is limited.

### 3.4 Carbon-based nanoparticles

Graphene oxide (GO) is a derivative of graphene, with a large number of oxygencontaining functional groups on its surface. Owing to its favourable physicochemical properties including good biocompatibility, large specific surface area conducive to biomolecule modification, high drug loading capacity, and relatively low toxicity, it has attracted the attention of researchers in the field of drug delivery (Wang B. et al., 2022). Wang et al. designed Fe3O4/GO NPs for the delivery of 5-fluorouracil (5-FU), utilising electrostatic interactions to coat GO onto the surface of hollow Fe3O4. Fe3O4/GO-5-FU reportedly shows no cytotoxicity within the concentration range of 2.5–40 μg/mL and exhibits controlled release capabilities; therefore, it may represent a promising potential anticancer drug (Wang et al., 2017). Interestingly, Lf is an iron-binding glycoprotein with a cation, and has proven to be non-toxic (Sabra and Agwa, 2020). Belonging to the Tf family of glycoproteins, Lf can bind to both LfR and TfR (Agwa and Sabra, 2021). Furthermore, LfR, akin to TfR, is widely expressed on the surface of brain endothelial cells (Agwa and Sabra, 2021). Moreover, Lf carries a positive charge, enabling dual-targeting of the tumour site through adsorption and receptor transcytosis, effectively enhancing the therapeutic effect (Elzoghby et al., 2020). Therefore, in order to further improve the therapeutic effect, Song et al. loaded the targeting ligand LF and superparamagnetic Fe3O4 NPs onto the surface of GO to form Lf@GO@Fe3O4 nanocomposite. In comparison to free DOX and DOX@GO@Fe3O4, Lf@GO@Fe3O4@DOX exhibited a higher inhibitory effect on tumour cell proliferation. Additionally, Lf@GO@Fe3O4@DOX demonstrated superior drug sustained release capability, possibly owing to the chemical interaction between DOX and Lf through hydrogen bonding (Song et al., 2017). Folic acid, a water-soluble vitamin B, plays a crucial role in cell proliferation and DNA synthesis (Ebrahimnejad et al., 2022). Tumour cells have folate receptors on their surface, enabling them to recognize and bind to folic acid; utilising this characteristic, surface modification of NPs with folic acid can facilitate their recognition and uptake by tumour cells. Interestingly, this approach has been proven feasible in studies targeting hepatocellular carcinomas, and breast and lung cancers (Hanafy et al., 2023a; Hanafy et al., 2023b; Ghazy and Hanafy, 2024). Therefore, this method may be a novel strategy to enhance the efficiency of glioma treatment. Wang et al. developed folate-modified graphene oxide (GO-FA) for the delivery of TMZ (GO-FA-TMZ). Their study found that GO-FA-TMZ exhibits controlled release of TMZ and pH-dependence. Moreover, *in vitro* experiments demonstrated significant inhibition of glioma cell growth by GO-FA-TMZ (Wang et al., 2020).

The application of GO as a nanocarrier for drug delivery is still in the early stages of research and has certain limitations. For instance, the distribution of GO in gliomas is not yet clear, and the safety of modified GO *in vivo* requires further confirmation. Additionally, the technology for drug release control is still developing (Wang B. et al., 2022). However, continuous exploration suggests that GO holds potential for applications in NDDS.

### 3.5 Inorganic nanoparticles

SiO2 (Silica) is one of the most abundant inorganic compounds in the world and has garnered widespread attention. Silica NPs (SiNPs) with uniform pore size, ease of surface modification, good biocompatibility, and stability have also been favoured by researchers (Huang et al., 2022). SiNPs can induce cellular damage in GBM, with a specific mechanism involving exposure of SiNPs leading to oxidative stress, subsequent mitochondrial dysfunction, and activation of inflammatory responses, ultimately causing cell death through apoptotic pathways (Kusaczuk et al., 2018). This suggests that SiNPs may represent a new therapeutic strategy; however, the specific distribution and safety of siNPs *in vivo* must be considered. SiNPs exposure can induce pathological features associated with Alzheimer’s disease, such as Aβ deposition and excessive tau protein phosphorylation, which could lead to irreversible harm to patients (Yang X. et al., 2014). Therefore, most of the current research has focused on targeting tumour tissues. Janjua et al. synthesised ultrasmall particles with large pore silica NPs (USLP) and conjugated them with Lf to load doxorubicin. *In vitro* experiments demonstrated that, compared to DOX alone and USLP-DOX, USLP-DOX-Lf enhanced the ability to cross the BBB and improved the anti-tumour therapeutic effect (Janjua et al., 2021a).

To optimise the rational application of SiNPs, it is essential to understand the methods of synthesising SiNPs and explore strategies for enhancing their targeting specificity while minimising their impact on normal tissues. These considerations are crucial for advancing future developments in this field.

### 3.6 Nanogel

NGs are among the most widely used NPs for drug delivery, particularly for temporal control. Notably, NG combines the advantages of hydrogels and NPs, including high biocompatibility, excellent drug-loading capacity, and responsiveness to environmental stimuli (Zhang H. et al., 2016). Therefore, NGs are ideal drug carriers.

Zhang et al. developed an Lf/phenylboronic acid (PBA)-functionalized hyaluronic acid NGs, referred to as Lf-DOX/PBNG, to deliver DOX for the targeted treatment of GBM. *In vitro* transport across the BBB model revealed that compared to DOX/NG and DOX/PBNG, Lf-DOX/PBNG exhibited stronger cytotoxicity, higher cell uptake efficiency, and enhanced brain permeability, leading to an improved area under the curve and biosafety of DOX (Zhang M. et al., 2021). Another study developed a biomimetic NG loaded with TMZ and the photosensitizer indocyanine green (ICG), which was surface-modified with a red blood cell membrane (ARNGs@TMZ/ICG) (Zhang et al., 2022). Under light exposure, ICG generates reactive oxygen species (ROS), leading to the degradation of ARNGs@TMZ/ICG and the release of drugs, thereby achieving therapeutic effects. These results indicate that this biomimetic NG can prolong the half-life of the drug, enhance its tumour-targeting effects, and consequently extend median survival.

NGs also have limitations, such as immunogenicity and colloidal instability. Therefore, continuous optimisation is necessary for clinical applications.

### 3.7 Cell-based nanoparticles

Leukocytes play a crucial role in the body’s defence response. There are various types of leukocytes, including neutrophils (NEs), eosinophils, basophils, monocytes, and lymphocytes, depending on their form, function, and source. The first three cells are collectively referred to as granulocytes (Stock and Hoffman, 2000; Angelillo-Scherrer, 2012; Kameritsch and Renkawitz, 2020). Our main focus was on NEs, which comprise more than half of the leukocytes in the blood (Friedmann-Morvinski and Hambardzumyan, 2023). During inflammation or tumour formation, chemokines recruit NEs, which then bind to chemokine receptors and subsequently to endothelial cells, allowing them to adhere. This process facilitates the migration of NEs through the spaces between endothelial cells to reach the sites of inflammation or tumours (Wang H. et al., 2021). For instance, GBM can produce IL-8 and attract NEs to tumour sites (Wang G. et al., 2022). Additionally, creating an inflammatory microenvironment through strategies such as surgery, radiation, and photothermal therapy can enhance chemokine recruitment, thereby improving the targeting ability of NEs (Wang H. et al., 2021). In summary, these findings highlighted the potential of NE-mediated NP drug delivery.

Xue et al. used NEs to transport cationic Lips (CL) containing PTX (PTX-CL/NEs). Subsequently, the PTX-CL/NEs effectively penetrated the BBB and inhibited the growth of GBM cells. Although this approach did not completely eradicate GBM in mice after surgery, it reduced or delayed glioma recurrence, thereby improving the survival rates. GBM recurrence may have been owing to the development of resistance to PTX or the presence of escaped tumour cells (Xue et al., 2017). Li et al. developed a nanosensitizer delivered by NEs, consisting of a core composed of ZnGa2O4:Cr3+ (ZGO) with the ability to emit persistent luminescence, and a shell composed of hollow sono-sensitive TiO2, which could enhance the production of ROS under insonation to control drug release. The anti-PD-1 antibody and PTX-loaded Lip (ALP) were encapsulated to form ZGO@TiO2@ALP. The results demonstrated that the ZGO@TiO2@ALP-NEs exhibited the strongest penetration ability, with the drug concentration targeting tumour cells reaching 35.6% (compared with 3.6% for PTX and 5.2% for ZGO@TiO2@ALP). This is attributed to the ability of ZGO@TiO2@ALP to induce local inflammation in the tumour environment and attract a large number of NEs. Furthermore, this method increased the survival rate of mice by 40% and significantly improved the therapeutic efficacy (Li Y. et al., 2021). Additionally, exosomes secreted by NEs can serve as an integral component of NEs, exhibiting strong inflammatory chemotaxis and the ability to traverse the BBB for the treatment of brain diseases, such as glioma (Wang H. et al., 2021). Wang et al. developed a system for GBM treatment using NE-derived exosomes (NEs-Exos) loaded with DOX. Experimental results showed that NEs-Exos effectively inhibited glioma growth in mice and prolonged their survival following intravenous injection. NEs-Exos also exhibit the same chemotaxis as NEs under inflammatory stimulation and successfully cross the BBB for tumour targeting (Wang J. et al., 2021).

The use of NEs as carriers is a novel concept; however, there are concerns regarding the potential for chronic inflammation following the injection of a large quantity of NEs (Hosseinalizadeh et al., 2022). Moreover, the extraction and purification of NEs from patients is invasive and may not be suitable for all patients requiring treatment (Hosseinalizadeh et al., 2022). Notably, the inflammatory tumour microenvironment has been implicated in the progression of low-grade to highly malignant GBM (Basheer et al., 2021). Therefore, a more rational and systematic design is required to address safety and feasibility concerns.

### 3.8 Virus-based nanoparticles

AAV is a non-enveloped, single-stranded DNA virus with a 4.7 kb genome enclosed in an icosahedral capsid (Issa et al., 2023). It contains two flanked inverted terminal repeats and two genes encoded by the genome: the rep gene responsible for DNA replication and packaging, and the cap gene coding for the capsid protein (Sabatino et al., 2022). Interestingly, AAV has been detected in various organisms but has not been associated with any disease (Issa et al., 2023; Li et al., 2023), suggesting its non-pathogenic nature. Consequently, AAV has gained popularity among researchers, with three AAV-based gene therapy drugs approved by the FDA in 2022 (Blair, 2022; Keam, 2022; Heo, 2023). Currently, there are 13 known serotypes of AAV (AAV1 to AAV13), with AAV2, 5, 8, and 9 being the most prevalent in clinical studies (Young, 2023). Furthermore, different AAVs demonstrate distinct tissue tropisms (Pupo et al., 2022), allowing the selection of brain targeting AAVs as carriers to enhance delivery efficiency.

AAVs have been used to treat gliomas for many years. Initially, owing to the presence of the BBB, therapy primarily involved local injections, which resulted in reduced local tumour growth (Zhong et al., 2017). The BBB-crossing function of AAV9 was discovered in 2009, leading to the adoption of systemic injections for the treatment of glioma (Foust et al., 2009). This noninvasive approach has shown broad efficacy in inhibiting glioma growth at various brain locations (Silva-Pinheiro et al., 2020), offering advantages over local injections. Similarly, AAVrh.8 and AAVrh.10 have also been found to exhibit BBB-crossing effects (Yang B. et al., 2014). In 2016, Crommentuijn et al. employed AAV9 as a carrier to deliver sTRAIL to treat gliomas via systemic injection for the first time (Crommentuijn et al., 2016). These findings confirmed that sTRAIL alone could not penetrate the BBB. However, when loaded onto AAV9, it successfully crossed the BBB, targeted GBM in the brain, slowed tumour growth, and significantly improved survival (Crommentuijn et al., 2016). Another study demonstrated that systemic injection of AAV9-IFN-β was more effective in inducing regression of multifocal GBM compared to local injection (GuhaSarkar et al., 2016).

However, systemic injection of AAV-based therapies presents numerous challenges; the foremost among these is the immune response (Weber, 2021). However, the presence of AAV antibodies in the human body severely limits the effectiveness of AAV gene therapy. Additionally, the capacity to cross the BBB and the transduction efficiency are significant concerns. Although AAV9 exhibits good BBB-crossing ability and transduction efficiency, it is not the most potent. Studies have indicated that AAV9 variants, such as AAV-PHP.B, AAV-PHP.eB, and AAV.CPP.16, possess stronger BBB crossing ability and transduction efficiency than those of AAV9 (Deverman et al., 2016; Chan et al., 2017; Yao et al., 2022). Furthermore, the broad transduction effect of AAV lacks specificity, making it prone to failure in achieving optimal therapeutic doses at the target and causing high toxicity to peripheral cells. Therefore, reducing the immune response, enhancing the BBB-crossing ability, and improving transduction specificity are crucial areas for future research.

## 4 Clinical perspectives

After years of experimental research, NP-based carrier-delivery technology has been successfully translated into clinical investigations for certain diseases. Currently, it encompasses Lips, red blood cells, mesenchymal stem cells, AAV, and other carriers. Drawing from the aforementioned carriers, we compiled a summary of existing clinical trials for the treatment of gliomas (Table 2).

**TABLE 2:** Representative clinical trials of various nanoparticle drug carriers.

Nanoparticle drug carriers	Approach	Interventions	Diseases	Phase	Number of participants enrolled (with or without a control group)	Age	Toxicity	Response rates and overall Survival	Identifier/DOI
Lip	Intravenous injection	Caelyx	Recurrent HGG	II	13; Without a control group	18-65	1. Palmoplantar erythrodysesthesia (38%); 2. Myelotoxicity (31%); 3. Minor side effects (31%)	No CR or PR	10.1002/1097-0142(20011001)92:7<1936::aid-cncr1712>3.0.co;2-h
		Caelyx with TMZ			22; Without a control group	31-80	1. Thrombocytopenia (18%); 2. Neutropenia (18%); 3. Rash/dry skin (45%); 4. Palmar-plantar changes (28%); 5. Nausea/vomiting (45%); 6. Mucositis (50%); 7. Reaction to Caelyx (18%); 8. Lethargy (59%)	CR (5%); PR (14%); SD (50%); PFS-6 (32%)	10.1215/S1152851703000188
		PEG-DOX with and without TAM			20 (PEG-DOX with TAM); 20 (PEG-DOX without TAM);	18-70	1. Palmoplantar erythrodysesthesia (25%); 2. Mucositis (7.5%); 3. Myelotoxicity (7.5%)	CR (2.5%); PR (2.5%); SD (35%); PFS-6 (15%)	10.1002/cncr.20073
		PEG-DOX with TMZ	Recurrent HGG	I/II	63; Control group (EORTC/NCI-C/NCI-C: 287)	18-70	1. Gastrointestinal: Vomitus/nausea (6.3%), Stomatitis (3.2%); 2. Palmoplantar erythrodysesthesia (6.3%); 3. Infection: Pneumonia (14.3%); 4. Blood/Bone marrow: Leukopenia (19%), Lymphopenia (62.4%), Thrombopenia (11.1%), Anemia (3.2%); 5. Cardiac and vascular toxicity: Deep vein thrombosis (3.2%), Pulmonary embolism (1.6%);	CR (3.2%); PR (4.8%); SD (65.1%); PFS-12 (30.2%)	NCT00944801
			GBM multiforme	II	40; Without a control group	24-76	1. Neutropenia (7.5%); 2. Hand-foot syndrome (5%); 3. Mucositis (2.5%); 4. Reaction to PEG-DOX (2.5%)	CR (3%); PR (0%); SD (82%); PFS-6 (58%)	10.1016/j.jocn. 2011.02.026
		nal-IRI	Recurrent HGG	I	16 (WT cohort); 18 (HT cohort)	WT: 28-65; HT: 28-75	1. Neutropenia (5.9%); 2. Leukopenia (17.6%); 3. Lymphopenia (14.7%); 4. Fatigue (asthenia, lethargy, malaise) (5.9%); 5. Nausea (2.9%); 6. Dehydration (8.8%); 7. Diarrhea (8.8%); 8. Elevation of creatinine (2.9%); 9. Hypokalemia (2.9%);	CR, PR, SD are not reported; PFS-6 (2.9%)	NCT00734682
		nal-IRI with TMZ	Recurrent GBM		12; Without a control group	39-70	1. Neutropenia (8.3%); 2. Diarrhea (16%); 3. Hypokalemia fatigue (8.3%); 4. Anorexia (16%)	CR, PR, SD are not reported; The median PFS was 2 months	NCT03119064
		LC with TMZ	HGG	I/II	30; Without a control group	≥18	—	—	NCT05768919

Abbreviation: Lip, Liposome; Caelyx, Liposomal Doxorubicin; CR, complete response; PR, partial response; SD, stable disease; PFS-6, The 6-month progression-free survival; TMZ, temozolomide; PEG-DOX, pegylated liposomal doxorubicin; TAM, tamoxifen; HGG, High-Grade Glioma; PFS-12, The 12-month progression-free survival; nal-IRI, nanoliposomal irinotecan; GBM, glioblastoma; LC, Liposomal Curcumin.

Lip-based NPs are the initial delivery carriers used in clinical trials. Clinical trials involving Lips have primarily focused on 1) PEGylated liposomes enhance BBB penetration, and PEGylation improves the stability of Lip and prolongs its pharmacokinetics, and 2) combining PEGylated Lips with two chemotherapy drugs to further improve patient survival. In a phase II clinical trial, PEGylated liposomal doxorubicin (Caelyx) demonstrated positive outcomes in patients with recurrent high-grade glioma (Fabel et al., 2001). Specifically, the group treated with Caelyx showed a longer median overall survival (OS) of 40 weeks than the other second-line chemotherapy options. Additionally, patients treated with Caelyx showed greater disease stability. However, no partial response (PR) or complete response (CR) was observed in this trial. In another phase II clinical trial, TMZ was combined with Caelyx for the treatment of recurrent glioma (Chua SL et al., 2004). The interaction between TMZ and Caelyx lies in their activity against different types of tumours, their formulation allowing prolonged drug exposure, and their lack of significant overlapping toxicity (Awada et al., 2004). Trial data indicated that this combination may offer advantages in terms of disease stabilisation and progression-free survival (PFS). However, there was no statistically significant difference compared to the efficacy of TMZ alone. Furthermore, the trial was terminated prematurely because it did not achieve the required objective response rate (CR + PR) specified in the study design. In a subsequent phase II clinical trial, Hau et al. introduced PEGylated doxorubicin Lips (PEG-DOX) for the first time, with or without Tamoxifen (TAM), to assess its efficacy (Hau et al., 2004). DOX can induce increased expression of P-glycoprotein and multi-drug resistance protein, leading to increased drug resistance in tumour cells (Abe et al., 1998). TAM has been found to be a resistance modulator that can inhibit the growth of glioblastoma without inducing resistance (Pollack et al., 1990). Therefore, combining the two in treatment may improve therapeutic efficacy. Trial data revealed that 40% of patients with grade III gliomas and 5% of those with grade IV gliomas experienced tumour progression-free periods of up to 160 weeks. The patients’ health status showed no discernible differences before and after treatment, indicating that combination therapy is an effective and safe treatment option. However, there were no significant differences in the PFS, median time to progression (TTP), or median OS between PEG-DOX combined with or without TAM. Similarly, other clinical trials have consistently concluded that the combination of PEG-DOX with chemotherapeutic drugs does not yield additional clinical benefits in terms of PFS and OS (Beier et al., 2009; Ananda et al., 2011). Consequently, no further clinical trials were conducted in recent years. Two clinical trials of nanoliposome irinotecan (nal-IRI) have been conducted. The aim of one trial was to confirm the safety and feasibility of intravenous injections (NCT00734682). Another trial combined nal-IRI with TMZ, but no activity was observed during the interim analysis, leading to the termination of the study (NCT03119064). Notably, a phase I/II clinical trial evaluating lipid-encapsulated curcumin in combination with TMZ is currently recruiting participants and is expected to be completed by May 2027 (NCT05768919).

In the NPs discussed in this review, only Lips have undergone relevant clinical trials. The results have shown that NDDS can indeed improve patient survival rates to some extent. Furthermore, surface modifications can further address the limitations of NPs, such as enhancing stability and prolonging their pharmacokinetics, thereby extending patient survival even further. However, achieving disease remission remains a challenge. Additionally, combination chemotherapy aims to utilize drugs that act through different mechanisms to reduce the likelihood of developing drug-resistant cancer cells. Nevertheless, the trial results have not demonstrated any additional clinical benefits.

Furthermore, other mediated delivery techniques such as CS, NE, and AAV are still in the early stages of glioma treatment and have not yet progressed to clinical research. Nonetheless, as new technologies continue to develop, more drug delivery methods are anticipated to advance clinical research.

## 5 Challenges in the clinical translation of nanoparticle carriers for the treatment of glioma

Research on the use of NPs for drug delivery in glioblastoma treatment has been ongoing for decades. However, the number of NPs that can be translated into clinical applications remains limited. Here, we summarize the challenges that need to be addressed for clinical translation, which can be divided into two main categories. Firstly, regarding NPs: 1) Targeting efficiency. Improving the efficiency of NP delivery and drug therapy, while reducing damage to normal tissues or cells; 2) Safety. Expanding the therapeutic dose used in animal models to humans, which may increase the potential for side effects associated with high doses of NPs; 3) Dose and therapeutic effect. Reducing drug dosage while maintaining therapeutic efficacy, given the safety concerns; 4) Large-scale production and high costs. Small-scale synthesis of NPs for cell or animal models is achievable, but large-scale production may impact the properties and functions of the NPs, leading to irregular shapes and poor stability. In addition, The cost of using NPs for conventional patient treatment may be excessively high. Secondly, the biological barriers *in vivo*: 1) BBB. BBB has always been considered the greatest obstacle for drug entry into the brain; 2) Mononuclear phagocyte system (MPS) and renal clearance pathways. Studies have shown that NPs with a size below 12 nm are cleared by the kidneys, while those above 200 nm are cleared by MPS. Therefore, it is necessary to reduce the clearance of NPs by these two biological systems to increase the chances of NPs reaching tumour tissues.

## 6 Prospectives

Treatment of glioma continues to face significant challenges. On reviewing the developmental trajectory of glioma treatment in recent decades, numerous areas of interest have emerged; however, few breakthroughs have been made. The field is still accumulating quantitative changes before achieving qualitative changes, a process that may span decades or even centuries. Currently, the primary treatment for gliomas is surgical resection, combined with radiotherapy and chemotherapy. Despite this, there has been little improvement in the patient’s life expectancy. Consequently, there is an urgent need for effective treatments to prolong patient survival and enhance prognosis. Notably, numerous studies have demonstrated that NDDS is a promising strategy, whether it involves Lip, AAV, or other NPs. Different types of NPs can target the BBB through various mechanisms, including 1) Lip, GO, SiNP, and NG achieve BBB targeting via the EPR effect; 2) CS targets the BBB via the AMT; 3) Ferritin targets the BBB via the RMT; 4) NE is targeted to the BBB through recruitment by chemokines in the inflammatory microenvironment; 5) AAV may target the BBB via RMT or other mechanisms, although the specific mechanism is not yet clear. Moreover, there are currently many NPs available for selection, and it is important to fully understand their physicochemical properties. For example, Lips have wide-ranging applications and a structure similar to cell membranes, but they suffer from poor stability and low drug loading. GO has a high specific surface area and excellent drug loading capacity, but it still faces challenges related to *in vivo* toxicity and drug release. CS has low production costs, but its safety needs to be considered. NG have strong drug release capabilities but poor stability. Ferritin and SiNP have wide sources, but the former competes with endogenous proteins for binding, and the latter may cause damage to normal cells. NE and AAV have higher biocompatibility, but large-scale production is difficult and costly. Notably, various types of NPs have different limitations that can be addressed through surface modifications, including 1) enhancing targeted drug delivery to improve therapeutic efficacy, 2) improving the physicochemical properties of NPs such as stability and solubility, and 3) prolonging drug release time to enhance patient compliance (Xu et al., 2022). Moreover, researchers have enhanced the therapeutic efficacy of drugs by conjugating ligands (such as TF, LF, and FA) to the surface of NPs for site-specific drug delivery, as discussed in previous chapters. Similarly, peptides also show excellent properties in improving active targeting, such as active targeting peptides and CPPs. The relative details can be found in this review (Luiz et al., 2021). These approaches may help the NDDS evolve into valuable clinical tools in the future.

Despite the rapid development of NDDS, many challenges remain unaddressed. The vast majority of *in vivo* experiments have been conducted in mouse tumour models, which differ from those of clinical patients. This discrepancy can lead to differences between the preclinical efficacy of an NDDS and its efficacy in clinical trials, thereby reducing the success rate of clinical translation. Therefore, the development of human tumours using *in vitro* or *in vivo* models and their application in NDDS treatment research may be an effective approach to improve clinical translation. Furthermore, as technology advances and NPs become more complex, ensuring their safety poses numerous challenges. For example, Lf-modified nanoliposomes improve brain targeting and the therapeutic efficacy of drugs; however, these nanoliposomes contain hydrogenated soybean phosphatidylcholine, and their secondary metabolites may have adverse effects on the nervous system (Wang et al., 2023). Therefore, a thorough understanding of the physicochemical properties of nanomaterials and the development of simple NP drug delivery methods are important strategies. Notably, recent studies have shown that breaking the disulfide bonds in Lf to form unfolded Lf for drug loading is a feasible and safe strategy (Na et al., 2024). The ultimate goal of anti-tumour nanomedicines is to extend the OS time of patients while maintaining the stability of their condition and good quality of life, rather than solely focusing on the ability of NDDS to effectively enhance the concentration and safety of drugs in the brain.

## 7 Conclusion

NDDS is an exciting strategy that offers hope to patients with glioblastoma. Currently, there are few breakthroughs in clinical progress, but we believe that liposomes are the most promising NPs for clinical translation. Despite their limitations, improvements through surface modification and coupling with ligands and peptides offer the potential for curing glioma patients. With the continuous progress of nanotechnology and in-depth research by scientists, more NPs are expected to achieve clinical translation and overcome the limitations of current treatment options. Overall, the NDDS has promising prospects and will achieve qualitative changes in the future.
